# *Burkholderia fungorum* Septicemia

**DOI:** 10.3201/eid1107.041290

**Published:** 2005-07

**Authors:** G. Peter Gerrits, Corné Klaassen, Tom Coenye, Peter Vandamme, Jacques F. Meis

**Affiliations:** * Canisius-Wilhelmina Hospital, Nijmegen, the Netherlands;; † Sint Maartenskliniek, Ubbergen, the Netherlands;; ‡ University of Ghent, Ghent, Belgium

**Keywords:** Burkholderia fungorum, Burkholderia infections, Burkholderia cepacia complex, bacteremia, infection

## Abstract

We report the first case of community-acquired bacteremia with *Burkholderia fungorum*, a newly described member of the *Burkholderia cepacia* complex. A 9-year-old girl sought treatment with septic arthritis in her right knee and ankle with soft tissue involvement. Commercial identification systems did not identify the causative microorganism.

The genus *Burkholderia* contains >30 species, of which *Burkholderia pseudomallei*, *B*. *mallei*, and members of the *B*. *cepacia* complex are the most well known. The species *B. fungorum* was recently proposed for isolates recovered from the environment, and animal and human clinical samples (1,2). Here we describe the first case of community-acquired bacteremia with *B*. *fungorum* in a 9-year old girl with the clinical features of septic arthritis.

## Case Report

A previously healthy 9-year-old girl had pain, swelling, and redness of the right foot and ankle 4 days before hospital admission, and similar symptoms of the right knee, 2 days before admission. One day before admission, a temperature of 39°C developed. She and her family had no history of arthritis, rheumatic arthritis, or other autoimmune disorders.

On physical examination, she had a body temperature of 38.8°C. Except for some slight swelling, pain, and redness of the right ankle, no other abnormalities or suspected source of the fever was apparent. She did not allow pressure on the calcaneus, which was painful.

Laboratory investigations demonstrated a C-reactive protein level of 262 mg/L, an erythrocyte sedimentation rate of 125 mm in the first hour, and a leukocyte count of 12.6 × 10^9^/L with 9.1 × 10^9^/L neutrophils. Levels of serum electrolytes, creatinine, and hepatic enzymes were within normal limits. Tests for antinuclear antibodies and antineutrophilcytoplasmic antibodies were negative. Serologic results for cytomegalovirus, *Toxoplasma gondii*, *Borrelia burgdorferi*, and Epstein-Barr virus were unremarkable. Throat and feces samples were negative for any virus. Feces cultures were negative for *Salmonella*, *Shigella*, *Yersinia*, and *Campylobacter*.

Radiologic examination of the right lower leg demonstrated no abnormalities of knee, foot, and ankle. A magnetic resonance imaging scan of the right lower leg showed a modest fluid collection in the soft tissues between the calcaneus and fascia plantaris, but no signs of osteomyelitis. An echogram of the abdomen was unrevealing, and a computed tomographic scan of the cerebrum showed no signs of an intracranial or sinus infection.

A bone scintigraph demonstrated a slight asymmetric increased signal in the epiphysial disc of the femur at the right knee. Because osteomyelitis with soft tissue involvement was suspected, she was empirically treated with intravenous cefuroxime (800 mg 3 ×/day), which was continued when the blood cultures became positive with gram-negative rods (on the fourth day of admission). Defervescence was initially seen within 24 h, but her temperature rose again to 38.5°C on the day 3 and up to 40.5°C on the day 7 of antimicrobial therapy. Because the cultured gram-negative rod was susceptible to cefuroxime in vitro, antimicrobial treatment was not changed. C-reactive protein level initially diminished gradually to 162 mg/L, but increased to 310 mg/L. Several diagnostic efforts, including a cardiac echogram, electrocardiogram, and an ear, nose, and throat workup, were carried out to determine the focus of infection but without convincing results. Although the patient had a clinical course of recurrent fever, she was otherwise stable, and we therefore decided to discontinue cefuroxime on day 9. At that time, the gram-negative rods in blood cultures, obtained before and during cefuroxime treatment, were reported to be *B*. *fungorum*. Intravenous ciprofloxacin, 170 mg 2 ×/day (15 mg/kg/day), was started; within 24 h, she became afebrile, and the C-reactive protein level became normal within 1 week. After 10 days, intravenous administration of ciprofloxacin was changed to oral administration (180 mg 2 ×/day) and continued for another 4 weeks. She recovered completely without sequelae.

On 3 following days, before and during treatment with cefuroxime, 5 blood cultures were collected in BACTEC aerobic pediatric resin bottles (Becton Dickinson, Sparks, MD, USA) and incubated in the BACTEC 9240 (Becton Dickinson). All blood cultures yielded gram-negative rods after 4 days of incubation. Subcultures produced a fine growth after 1 day of incubation.

The organism was positive for catalase and oxidase, and routine identification procedures with the API 20NE kit and database release 6.0 (bioMérieux, Marcy l'Etoile, France) produced the numerical code profile 1066157 which, according to the database, was a good identification (98.9% T 0.42) as *B*. *cepacia*. Antimicrobial susceptibility testing was performed with broth microdilution in accordance with Clinical and Laboratory Standards Institute (formerly NCCLS) protocols (3). The organism was susceptible to amoxicillin (MIC 1 mg/L), cefotaxime (MIC 0.5 mg/L), ceftazidime (MIC <0.5 mg/L), cefuroxime (MIC 1 mg/L), trimethoprim/sulfamethoxazole (MIC <1 mg/L), meropenem (MIC 0.25 mg/L), tobramycin (MIC <0.25 mg/L), and ciprofloxacin (MIC 0.5 mg/L), but resistant to cefazolin (MIC >32 mg/L). This rather susceptible microorganism prompted us to repeat the identification with partial sequencing of the 16S rRNA gene. DNA was isolated from a pure culture by using established protocols (4). Amplification primers 5´-CCTAACACATGCAAGTCGARCG-3´ (forward) and 5´-CGTATTACCGCGGCTGCT-3´ (reverse), both from Eurogentec (Seraing, Belgium) were used in a standard polymerase chain reaction (PCR) to generate a 490-bp fragment from the 5´ end of the 16S gene. The PCR (25 μL) consisted of 1 μL DNA, 0.5 μmol/L of both PCR primers, 1.5 mmol/L MgCl_2_, 0.2 mmol/L dNTP, and 1 U FastStart Taq DNA polymerase (Roche Diagnostics, Almere, the Netherlands) in 1 × reaction buffer. Cycling conditions were as follows: 30 s at 94°C, 30 s at 56°C, and 1 min at 72°C, repeated 30 times, preceded by a 10-min activation step at 94°C and followed by an additional 10 min elongation step at 72°C. The obtained amplicon was purified by using High Pure chemistry (Roche Diagnostics) and sequenced with the reverse amplification primer using a MegaBACE DYEnamic ET Dye terminator Kit as suggested by the manufacturer (Amersham Biosciences, Roosendaal, the Netherlands). Reaction products were purified by ethanol precipitation, dissolved in distilled water, and analyzed on a MegaBACE 500-capillary DNA analysis platform (Amersham Biosciences) under standard electrophoretic conditions. The obtained DNA sequence was compared to the public DNA databases by using the BLAST interface (http://www.ncbi.nlm.nih.gov/BLAST/) (5) and proved to be 100% identical to previously reported *B*. *fungorum* sequences.

To confirm this molecular identification, cellular protein and fatty acid analyses were also performed. Whole-cell proteins were prepared and evaluated with sodium dodecyl sulfate–polyacrylamide gel electrophoresis (1). The identification of the isolate as *B*. *fungorum* was subsequently confirmed by comparing it to a large database, which contained profiles of all *Burkholderia*, *Ralstonia*, and *Pandoraea* species and various other gram-negative nonfermenters (2). After aerobic growth for 24 h on Trypticase soy agar (Becton Dickinson, Erembodegem, Belgium), a loopful of well-grown cells was harvested, and fatty acid methyl esters were prepared, separated, and identified by using the Microbial Identification System (MIDI, Inc., Newark, DE, USA). By using the commercially available database (MIDI, Inc.), the isolate was again falsely identified as *B*. *cepacia* with a score of 0.611.

The oxidase-positive isolate reduced nitrate and assimilated glucose, mannose, mannitol, N-acetyl-glucosamine, adipate, malate, citrate, and caprate. Beta-galactosidase activity was present.

## Conclusions

To the best of our knowledge, this case is the first description of bacteremia and invasive infection due to *B*. *fungorum*. The name *B. fungorum* was recently proposed for a group of 9 *B. cepacia*–like isolates recovered from the environment and human and animal clinical samples (1). The only 2 strains from human clinical samples in that study were recovered from the vaginal secretions of a pregnant woman (22 weeks) with *Candida* sp. vaginitis and preterm labor, and the cerebrospinal fluid of a 66-year-old woman, respectively. No clinical data were available from these patients; therefore, the clinical significance could not be determined. Since the original report was made, *B*. *fungorum* has been identified in a range of soil- and plant-associated samples, in infections of the central nervous system of a pig and a deer (H. Scholz and P. Vandamme, unpub. data), and in the respiratory secretions of people with cystic fibrosis (1,2). However, in these cases, the clinical significance of isolation of *B*. *fungorum* was also unclear.

Since *B*. *fungorum* was only described recently and has not been found frequently in clinical samples, the organism is not included in most commercial biochemical databases used for identification, and it has previously been shown that *B. fungorum* isolates can easily be misidentified as *B*. *cepacia* complex organisms (1,2). A similar misidentification with conventional commercial biochemical tests was recently described in a case of *B*. *cenocepacia* vaginal infection (6). In the present case, the antimicrobial susceptibility profile, the patient's history, and the clinical findings suggested that this isolate did not belong to the *B*. *cepacia* complex, and the identification as *B*. *fungorum* was confirmed by using a polyphasic approach. Most *B*. *cepacia* complex infections in non–cystic fibrosis patients are nosocomial in origin, but severe community-acquired infections (including endocarditis, brain abscesses, and pneumonia) have also been reported (7). As this case illustrates, *B*. *fungorum* may pose a challenge to many clinical microbiology laboratories, and infections with this organism may be erroneously diagnosed as *B*. *cepacia* complex infections. A complete immunologic workup for our patient did not give any indication of why this child experienced this infection. The source of the *B*. *fungorum* bacteremia in our patient remained elusive, but, undoubtedly, it was a community-acquired infection manifested as a soft tissue infection of her leg. Of interest was the clinical failure of cefuroxime therapy, despite the isolate's in vitro susceptibility, and the rapid response to ciprofloxacin treatment.
